# Selection of Optimal Cell Lines for High-Content Phenotypic Screening

**DOI:** 10.1021/acschembio.2c00878

**Published:** 2023-03-15

**Authors:** Louise Heinrich, Karl Kumbier, Li Li, Steven J. Altschuler, Lani F. Wu

**Affiliations:** † Department of Pharmaceutical Chemistry, University of California San Francisco, San Fancisco, California 94158, United States

## Abstract

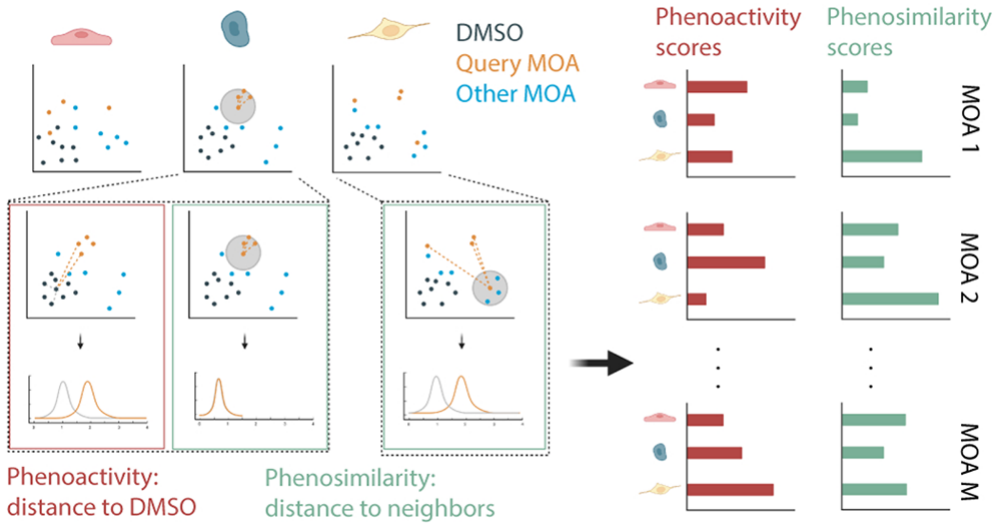

High-content microscopy offers a scalable approach to screen against multiple targets in a single pass. Prior work has focused on methods to select “optimal” cellular readouts in microscopy screens. However, methods to select optimal cell line models have garnered much less attention. Here, we provide a roadmap for how to select the cell line or lines that are best suited to identify bioactive compounds and their mechanism of action (MOA). We test our approach on compounds targeting cancer-relevant pathways, ranking cell lines in two tasks: detecting compound activity (“phenoactivity”) and grouping compounds with similar MOA by similar phenotype (“phenosimilarity”). Evaluating six cell lines across 3214 well-annotated compounds, we show that optimal cell line selection depends on both the task of interest (e.g., detecting phenoactivity vs inferring phenosimilarity) and distribution of MOAs within the compound library. Given a task of interest and a set of compounds, we provide a systematic framework for choosing optimal cell line(s). Our framework can be used to reduce the number of cell lines required to identify hits within a compound library and help accelerate the pace of early drug discovery.

## Introduction

Large, diverse libraries of novel small molecules serve as critical components in the early drug discovery screening pipeline.^1^ The cellular activities of the compounds in these libraries are typically unknown. High-content microscopy is a scalable approach for characterizing the effect of small molecules on cells.^2−5^ In this setting, cellular responses to compounds are represented as feature vectors, whose entries measure observable changes, such as in cell morphology, biomarker intensity, and localization. A single-pass phenotypic profiling screen can provide annotation to compound libraries by identifying subsets of library compounds that show “phenoactivity” (i.e., induce cellular responses distinct from control conditions) and by inferring MOA through “phenosimilarity” (i.e., by comparing cellular responses of compounds annotated with the same MOA).^6−9^

Phenotypic profiling screens depend critically on the selection of cellular readouts and screened cell lines. For the purposes of annotating large compound libraries, previous work has focused on selection of “optimal”^10^ as well as general^11^ combinations of cellular readouts for a given cell line. However, approaches for selecting optimal cell lines are less explored.^12−14^ It is reasonable to expect that different cell lines may have different sensitivities to detect different MOAs. For instance, others have focused on identifying compounds that induce differential responses across multiple cell lines.^15,16^ However, it is poorly understood how to select the best performing cell line for annotating diverse compound libraries in an unbiased- or target-agnostic fashion, and to what extent using multiple cell lines would improve coverage.

To explore these questions, we first generated a high-content microscopy data set of six cell lines responding to a diverse set of 3214 small molecules with annotated MOA. We developed computational methods to rank cell lines and combinations of cell lines according to their ability to infer compound activity and MOA. Lastly, we applied a classic optimization framework to assess which cell lines or cell line combinations would be best for annotating uncharacterized compounds.

## Results

### Experimental Design

Our data set was generated using six cell lines. These included five cancer cell lines (A549, OVCAR4, DU145, 786-O, HEPG2) from the NCI60 set of tumor cell lines; these cell lines span a range of tissue types (including epithelial, endothelial, neuronal, secretory (ductal), monoblast and erythroid origins), cellular morphologies, and are amenable to imaging assays. We additionally included a noncancer patient-derived fibroblast cell line (“FB”). Additional details in Supporting Information.

We collected a “reference” library of 3214 compounds annotated with mechanism of action (MOA). This library includes FDA-approved drugs, in-clinical-trial drugs, and established bioactive tool compounds. MOA annotations were curated from the Drug Repurposing Hub database and serve as an independent validation of our phenotypic profiling methods. Compounds cover 664 MOAs (Supporting Information Table S1) in both cancer-related pathways as well as the broader druggable target space. Due to the number of cell lines and drugs, we limited data collection to 48 h, and a single dose (either 5 or 10 μM, depending on the library used) treatment. Large screens of diverse compound sets are likely to include compounds that act over different time frames; we previously observed that the 48 h timepoint provided high classification accuracy on reference drug classes and overall diversity of phenotypic responses.^10^

To label cells, we made use of the Cell Painting assay^11^ and sampled nine fields of view at 20× magnification for each well by microscopy. For each treatment, we performed cell segmentation and feature extraction to generate 77 quantitative features (Supporting Information Table S2) describing cellular morphology and the distribution (i.e., intensity, texture, objects) of the six intracellular stains that comprise the Cell Painting marker set. We generated phenotypic profiles that summarized the population-level shift in each feature from a negative control condition (DMSO) using signed KS statistics^7^ (Supporting Information). Thus, the cellular response to each compound perturbation was summarized as a 77-dimension profile in phenotypic space.

### Data Analysis

Using these data, we sought to identify “optimal” cell lines or combinations of cell lines using two measures of annotation performance on the reference compound library: (1) *phenoactivity*: the degree to which compound and DMSO profiles differ; and (2) *phenosimilarity*: the degree to which the compounds with the same MOA share similar phenotypic profiles (Supporting Information).

Our approach focused on three key challenges. First, quantification of phenoactivity and phenosimilarity depend on analytical parameters, which can be challenging to tune across multiple cell lines. We aimed to limit the number of such parameters and ensure that results were robust to these parameter choices. Second, cellular phenotypic changes from DMSO control can range from subtle to severe across different cell lines. We aimed to ensure that our measurements of phenoactivity and phenosimilarity were sensitive across this spectrum and could report on responses that were similar to, but distinct from control. Third, compounds annotated to share a common MOA may induce phenotypically dissimilar responses (e.g., due to different on- or off-target activities, potency, coarse annotation, and so on). We aimed to define “phenotypic tightness” of an MOA in a manner that was robust to some degree of compound heterogeneity.

Based on these requirements, we chose to define phenoactivity and phenosimilarity at the MOA level rather than at a compound-by-compound level. In brief, for each cell and MOA, we computed: (1) a phenoactivity score by comparing the distributions of distances of the MOA and DMSO point clouds to the centroid of the DMSO point cloud; and (2) a phenosimilarity score by comparing the tightness of the MOA point cloud relative to the nearest neighbor point clouds of each MOA compound.

### Phenoactivity

**Figure 1:**
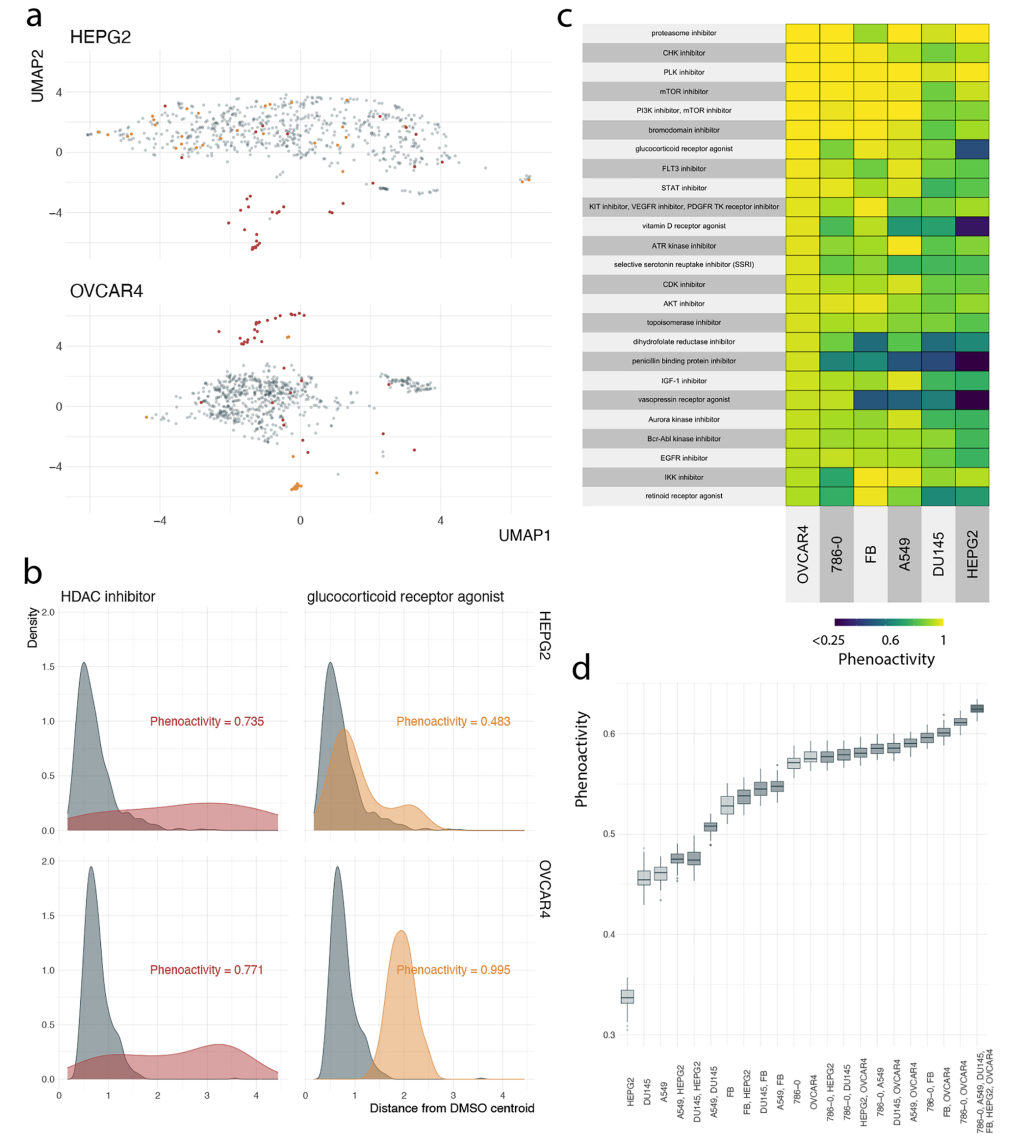
Optimizing cell line selection for phenoactivity. (a) UMAP projection of phenotypic profiles for query MOA samples (HEPG2 top, OVCAR4 bottom) and DMSO. (b) Distribution of distances to the DMSO point cloud centroid for DMSO samples and query MOAs by cell line (HEPG2 top, OVCAR4 bottom). Query distributions that are further from DMSO reference result in higher phenoactivity scores. (a-b) colors: compound MOA (red = HDAC inhibitor, yellow = gluccocorticoid receptor agonist). (c) Phenoactivity scores by cell line (column), MOA (row). MOAs are filtered to the top 25 scoring categories in OVCAR4 with at least 5 compounds. (d) Distribution of phenoactivity scores by cell line set, evaluated over 50 random subsamples of the library (2/3 of compounds subsampled). Color: number of cell lines from 1 (lightest) to 6 (darkest).

We first assessed the ability of individual cell lines to detect phenoactivity of compounds from specific MOA classes (Supporting Information Table S3). In all cell lines, positive control compounds were phenotypically distinct from DMSO controls (Figure S4). We visualized how compound profiles were distributed in phenotypic space for different cell lines using UMAP.^17^ In some cases, point clouds for collections of compounds annotated with the same MOA and DMSO replicates were similarly separated across cell lines, while in others dramatic cell line differences were apparent. For example, in the case of the HDAC inhibitors, both HEPG2 and OVCAR4 cell lines showed similar degrees of separation between HDACs and DMSO, with both cell lines detecting phenoactivity for a similar subset of HDAC compounds (e.g., 28/35 and 29/35 of HDAC compounds fell outside the DMSO point cloud—were more than one IQR above the median DMSO to DMSO centroid distance—in OVCAR4 and HEPG2 respectively; Figure 1a, top). In contrast, in the case of glucocorticoid receptor agonists (GRA), for OVCAR4 all GRA compounds were outside the DMSO cloud while a minority were for HEPG2 (29/29 vs 11/29 for OVCAR4 vs HEPG2, respectively; Figure 1a, bottom). These observations were consistent with our phenoactivity scores based on conversion of these point clouds into distance distributions (Figure 1b). While OVCAR4 was overall the most sensitive for detecting phenoactivity, other cell lines performed better in 88/148 MOAs containing at least 5 compounds. We summarized phenoactivity scores of top-ranked MOAs for OVCAR4 to highlight categories with consistently high activity (Figure 1c). Phenoactivity scores for all cell-line-MOA pairs are provided in Supporting Information Table S3.

Interestingly, most MOAs showed low phenoactivity in HEPG2. To assess phenotypic features that best distinguished HEPG2 from other cell lines, we trained an iterative random forest^18^ to classify HEPG2 versus other cell lines treated with DMSO control (Supporting Information; Figure S5). The classifier achieved near perfect accuracy on a hold-out test set of well replicates (area under the receiver operating characteristic curve 0.999), suggesting that HEPG2 cells were systematically distinct from the remaining cell lines (Figure S5b). To identify features that drove this separation, we evaluated mean decrease in impurity (MDI) feature importance and found that the classifier was heavily influenced by cell nearest neighbor distance (Figure S5c). This finding was consistent with qualitative examination of cell images (Figure S5a), which highlighted HEPG2 cell’s tendency to clump closely together.

We hypothesize that the growth of HEPG2 in highly compact colonies underlies the poor performance of this cell line in producing phenotypic profiles that are able to distinguish compound-induced phenotypes from control. Several of the markers used in our study target cellular organelles (mitochondria, actin) that would be difficult to distinguish alterations for compact cells. In addition, overall morphology (geometry) features of the cell are an important driver of phenotypic variation, and HEPG2 is less variable in its geometry due to its colony growth pattern. This example of a poorly performing cell line highlights the importance of considering cell morphology when selecting a cell line of interest for phenotypic screening—and exemplifies the use of our framework to select-out cell lines with these properties.

### Phenoactivity Optimization

How much improvement in phenoactivity detection is provided by inclusion of additional cell lines? We compared the abilities of individual and pairs of cell lines to detect phenoactivity across all MOA categories (Figure 1d). For a pair of cell lines, we defined the phenoactivity of an MOA as the maximum phenoactivity over each individual line—effectively asking whether phenoactivity is detected in either cell line (Supporting Information). The single best performing cell line is OVCAR4. By construction, pairs of cell lines including OVCAR4 outperform OVCAR4 on its own. However, the improvements relative to OVCAR4 alone were marginal (∼6.1% increase from phenoactivity score of 0.576 for OVCAR4 to 0.611 for OVCAR4, 786-0).

### Phenosimilarity

**Figure 2:**
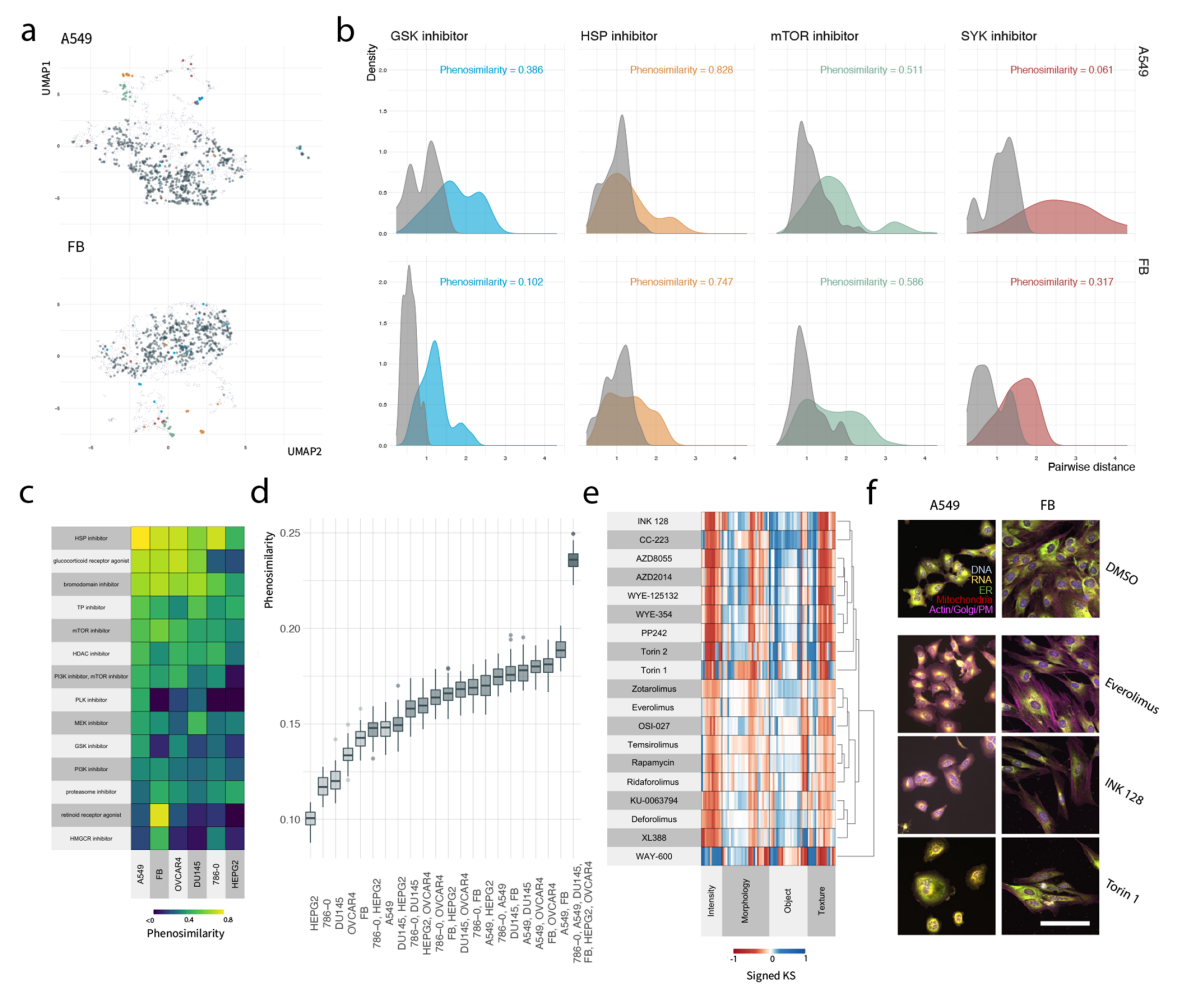
Optimizing cell line selection for phenosimilarity. (a) UMAP projection of phenotypic profiles by cell line (A549 top, FB bottom). (b) Distribution of pairwise distances between query MOA compounds (colored distribution) and their nearest neighbors (gray distribution) by cell line (A549 top, FB bottom). (a-b) Color: MOA (blue = GSK inhibitor, yellow = HSP inhibitor, green = mTOR inhibitor, red = SYK inhibitor, dark gray = DMSO; purple, small points = other MOA; light gray distribution corresponds to nearest neighbors of a given MOA class). (c) Phenosimilarity scores by cell line (column), MOA (row). MOAs are filtered to top 10 scoring categories in A549 and FB cell lines with at least 5 compounds. (d) Distribution of phenosimilarity scores by cell line set, evaluated over 50 random subsamples of the library (2/3 of compounds subsampled). Color: number of cell lines from 1 (lightest) to 6 (darkest). (e) Clustered phenotypic profiles for mTOR compounds evaluated in A549. (f) Representative images of compound treated cells (A549 or FB) after 48 h exposure to to DMSO vehicle control 0.1% (top) and select mTOR inhibitors (bottom 3). Scale bar represents 100 μm.

We next assessed whether compounds with the same provided MOA induce similar cellular phenotypes, which we quantified through phenosimilarity scores (Supporting Information Table S4). As previously, we assessed the ability of individual cell lines to detect phenosimilarity of specific MOA classes (Figure 2a-b). Here, we compared the distribution of distances among compounds within an MOA (colored distributions) to the distribution of those compounds’ nearest neighbors (gray distribution). In the case when an MOA is tightly clustered, the nearest neighbors of a compound will be another compound in the same MOA and the two distributions will closely overlap; conversely, if the point cloud of an MOA is broadly distributed and contains profiles of compounds in other MOAs, these distribution will look dissimilar. We note that the ability to detect phenosimilarity in a given cell line is dependent on the specific set of experimental parameters considered in our analysis (e.g., marker set, treatment time, treatment dose). Cellular heterogeneity may be driven in part by the fact that phenoactivity and phenosimilarity for a particular MOA/cell line combination cannot be detected with the parameters used in our analysis.

MOAs with high phenosimilarity in a given cell line were tightly clustered and fell further from the DMSO point cloud (Figure 2a). MOAs with low phenosimilarity across all cell lines were evenly distributed across the DMSO point cloud (Figure S6)—i.e. they could not be distinguished from DMSO controls. As a specific example, we compared MOA point clouds and phenosimilarity scores between the A549 and FB cell lines (Figure 2a-b). While HSP inhibitors were tightly clustered in both cell lines (phenosimilarity scores 0.828 and 0.747 in A549 and FB respectively), SYK inhibitors and glycogen synthase kinase (GSK) inhibitors showed varying degrees of differential clustering behavior between the two cell lines (SYK inhibitor phenosimilarity score 0.061 and 0.317 in A549 and FB respectively, GSK phenosimilarity score 0.386 and 0.102 in A549 and FB respectively).

Differential phenosimilarity of GSK3 inhibitors between A549 and FB can be explained by the distinct roles of GSK3 in these 2 cell lines. Nonsmall cell lung cancer cell lines, such as A549, exhibit increased GSK3 kinase activity, which supports tumor cell proliferation and contributes to the poor prognosis.^19,20^ Thus, GSK3 inhibition is expected to produce phenotypes in A549. Indeed, 8/11 GSK3 inhibitors showed phenoactivity in A549, and clustered closely together in phenotypic space (only SB216763, Tideglusib, and SB415286 clustered near DMSO). In contrast to A549, increased GSK3 kinase activity has not been reported in the noncancer FB cell line and GSK3 inhibitors showed low rates of phenoactivity and phenosimilarity.

Moderate phenosimilarity scores for mTOR inhibitors (0.511 A549, 0.586 FB) highlight the fact that while these compounds cluster near one another in phenotypic space, they closely neighbor other compounds with different MOA annotations. In the case of A549, a subset of closely grouped mTOR compounds is reflected in the bimodality of the distance distribution (Figure 2a-b). The two subclusters of mTOR inhibitors contain compounds with different inhibition mechanisms: one cluster is formed solely from mTOR kinase domain inhibitors that inhibit the activities of both mTORC1 and mTORC2, while the other cluster is formed mainly from allosteric inhibitors (rapamycin-analogues) that inhibit mTORC1 more selectively (Figure 2e). Qualitative examination of phenotypes revealed that the mTOR kinase domain inhibitor group induced a “clumping” phenotype (Figure 2f, INK 128 and Torin 1). This phenotype is characterized by features capturing distorted morphology (e.g., cell compactness, radial axis length, contact with neighbors, etc.; Figure 2e). In contrast, the allosteric inhibitor group showed less severe differences in morphology but induced changes in cellular objects (Figure 2e).

### Phenosimilarity Case Studies

To inspect whether cellular phenotypes induced by different MOAs were observable in images, we considered three well-studied categories: TP inhibitors, proteasome inhibitors, and PLK inhibitors. As caveats: we note that MOA classes are better separated by considering the entire phenotypic profile, rather than changes in any single feature; a single cellular change may be read out in multiple features (e.g., increases in puncta could result in changes to intensity and/or texture features); and some features may capture multiple effects observed in images (e.g., changes in cell shape could cause changes in the distribution of cytosolic markers). Nevertheless, these case studies provide an opportunity to examine whether cellular phenotypes corroborate prior work.

TP inhibitors that were distinct from DMSO clustered more closely in phenotypic space for A549 compared to FB, reflecting its slightly higher phenosimilarity score. Phenotypically, these compounds induced changes in morphology that can be interpreted as the result of cell cycle defects, observed in response to microtubule inhibition at longer time points (e.g., 48 h). This is a well-described phenotype resulting from microtubule inhibition in the literature.^21−23^ Specifically, upon treatment we observe fragmented nuclei and increases in cellular size in both cell lines (Figure S7b). These changes are reflected in decreases of nuclear roundness (Figure S7a, (i), the nuclear Hoechst (DNA) texture homogeneity signal (Figure S7a, (ii), and the cell compactness (Figure S7a, iii-iv).

Similar to TP inhibitors, polo-like kinases have pleiotropic roles in controlling cell cycle progression, in particular during the segregation of DNA by microtubules through its stabilizing activity at the kinetochore in prometaphase.^24^ Treatment with PLK inhibitors will often arrest cells in the G2/M phase, causing aberrant mitotic and postmitotic phenotypes and ultimately toxicity to fast-cycling cancer cell lines.^25^ Consistent with these findings, PLK inhibitors show strong phenotypes associated with cell cycle defects in both our A549 and FB, with cells that have progressed past G2/M displaying fragmented multiple small nuclei due to DNA segregation errors (Figure S8b). Ultimately, cells trapped in mitosis either aberrantly progress through or undergo apoptosis. As with TP inhibitors, these changes were reflected in changes to nuclear morphological (Figure S8a, i-ii) and texture features (Figure S8a, (iii).

The proteasome complex forms a key part of the ubiquitin-proteasome system, functioning as a machinery for the intracellular degradation of proteins, as part of protein turnover during proliferation, as well as maintenance of normal cellular homeostasis.^26^ The inhibition of the proteasome complex by proteasome inhibitors results in apoptosis, with cancer cells being particularly sensitive to this class of drugs.^27^ We observe a high level of cells undergoing early apoptosis in our assay associated with treatment with proteasome inhibitors, which is reflected by phenotypic changes such as a decrease in cell density and changes in cell morphology as the cells undergo apoptosis (Figure S9b). We observe the formation of both vacuolar and punctate structures across multiple channels in the cytoplasmic compartment, as well as a condensation of subcellular structures into bright puncta (particularly for MG-115). These changes are reflected in our phenotypic profiles by the observed reduction in cell contact area with neighbors (i.e., the cells are now sparsely populating the field; Figure S9a, (i), as well as a decrease in homogeneity of the signal (uniformity of a marker) in the DNA (Figure S9a, (ii), ER (Figure S9a, (iii), and Actin/Golgi/membrane channels (Figure S9a, (iv). The intensity of mitotracker also increased as the remaining mitochondria condense around the perinuclear region (Figure S9a-b).

### Phenosimilarity Optimization

Which cell line is the best “generalist” for grouping compounds based on these provided MOAs? We summarized phenosimilarity scores across all MOA-cell line pairs (Figure 2c). In this data set, A549 was the most sensitive for grouping compounds with the same MOA. We summarized phenosimilarity scores of top-ranked MOAs for A549 to highlight categories with consistent cellular phenotypes (Figure 2c). Phenosimilarity scores for all cell-line-MOA pairs are provided in Supporting Information Table S4. While A549 was the best “generalist”, there were 108/149 MOAs in which another cell line performed better. For screens focused on these specific MOAs, other cell lines may perform better as “specialists”.

How much improvement in MOA phenosimilarity is provided by inclusion of additional cell lines? We computed the MOA phenosimilarity across a pair of cell lines by taking the maximum score across individual lines—effectively asking whether an MOA is tightly clustered with respect to either cell line (Supporting Information). Here, the best performing cell line based on phenosimilarity is A549. Including an additional cell line with A549 offered a ∼28% improvement in average phenosimilarity score (0.148 for A549 vs 0.189 for A549, FB), driven by addition of several MOAs that were poorly clustered in A549 (e.g., FB improved identification of SYK, HMGCR, and proteasome inhibitors; Figure 2d).

## Discussion

A fundamental choice when setting up a large-scale high-content phenotypic screen is to determine which cell line or cell lines are best suited to identify bioactive compounds and predict their MOAs. Here, we provide a roadmap for how to objectively answer this question. We make use of an annotated reference compound library with hundreds of MOA classes to calibrate the performance of each potential screening cell line as well as combinations thereof. Phenoactivity scores can be used to select cell lines that best identify bioactive compounds. Phenosimilarity scores can be used to select cell lines that best identify compounds within specific MOA(s).

In our case study, we found that the best performing cell lines were different for either identifying bioactive compounds or categorizing compounds by MOA classes, and that the utility of using additional cell lines was not the same for each of these tasks. A cell line that is optimal to detect phenoactivity may have poor sensitivity for the same MOA class (and vice versa). This is because the ability to infer MOA from clusters in phenotypic space requires that compounds within the target MOA are near one another yet far from compounds with other MOAs. Phenosimilarity scores capture these two properties. In practice, large compound libraries could be annotated by evaluating how phenotypically similar unknown compounds are to known reference classes. Phenosimilarity scores assess the degree to which reference classes are phenotypically consistent and thus provide a measure of confidence for the degree to which new, phenotypically similar compounds belong to a given MOA.

There are a number of ways in which our approach could be improved or extended. From a platform perspective, we made a number of technical choices, including compound dose, treatment time, cell features, construction of phenotypic profile, distance metric, optimization framework, and so on. Each of these choices can be further examined for improved performance. From a more general perspective of using high-content imaging to detect and predict MOA of uncharacterized compounds, three are three major inputs to this process: cell line, biomarker, and annotated reference compound sets. Here, we optimized over cell lines while holding the biomarkers (cell painting) fixed, while in past work, we optimized over biomarkers while holding a cell line fixed.^10^ In both cases we assumed that a reference compound library was provided with MOA annotation classes. A future task is to investigate how phenosimilarity scores can be used to refine or coarsen provided MOA annotation classes. For instance, by replacing the DMSO reference distribution with some other MOA class in our phenoactivity analysis, one could evaluate the similarity between two MOA classes and provide guidance for when two MOA classes should be merged. Such analyses offer a new path to annotating compound libraries based on phenotypic consistency. A larger task, for future work, is an experimental and computational platform designed to optimize over all three input choices for a given screening goal.

High-content microscopy screens offer the promise to annotate large libraries of uncharacterized compounds. However, the ability of different cell models to classify compounds varies according to their underlying biology. Here, we show an objective framework for selecting one, or a small number of cell lines that are “optimal” for predicting bioactivities across specified MOA classes. It is our hope that this framework will help increase the scale, sensitivity, and accuracy of phenotypic profiling used in early drug discovery.

## Data Availability

Data and code to reproduce figures and analyses are available on Zenodo.

## Supporting Information Available

Annotated compound library (XLSX)
Computational features used to generate phenotypic profiles (XLSX)
Summary of results (XLS)
Summary of results (XLS)
Detailed description of experimental methods; supplemental figures (PDF)
