# Supplementary material for: Selection of Optimal Cell Lines for High-Content Phenotypic Screening
Source: ACS Chem Biol. 2023 Mar 15;18(4):679–85. doi: 10.1021/acschembio.2c00878 (PMC10127200; doi:10.1021/acschembio.2c00878)
Supplement: Supplementary file 5 — cb2c00878_si_005.pdf [file cb2c00878_si_005.pdf]

Supporting Information

Selection of optimal cell lines for high-content phenotypic screening

Louise Heinrich,[†,‡] Karl Kumbier,[†,‡] Li Li,[†] Steven J. Altschuler,[*,†] and Lani F. Wu[*,†]

†*Department of Pharmaceutical Chemistry, University of California San Francisco, San Francisco, California, 94158, United States*

‡*L.H. and K.K. contributed equally to this work*

E-mail: steven.altschuler@ucsf.edu; lani.wu@ucsf.edu

Materials and methods

Compound library

All screening plates were treated with 28 wells of vehicle (DMSO), and 4 wells containing positive controls (Bortezomib 5uM, Gemcitabine 5uM). Compound libraries were sourced from Selleck Chemicals LLC: FDA-approved & Passed Phase I Drug Library (L3800) (10uM), Kinase Inhibitor Library (L1200)(10uM), Apoptosis Compound Library (L3300)(10uM), Epigenetics Compound Library (L1900) (10uM),and Bioactive Library (L1700)(5uM). In addition to these compound libraries, in each screening batch we also added two replicate plates containing a "Reference Set" of 35 compounds, each present at 5 5-fold dilutions as described in.[1]

Compound MOA annotation

Screened compounds with the same chemical structure (SMILES and InChIKey) are combined under the same common name (Compound_ID). MOA annotations are curated from the Drug Repurposing Hub database and mapped to each compound based on their chemical structures. For a full list of compounds screened and annotations assigned (Supporting Information Table 1).

Cell culture

We cultured a subset of the NCI60 database, including A549, 786-O, OVCAR4, DU145 and HEPG2. In addition, we added a healthy fibroblast line "FB". A549, 786-O, OVCAR4, DU145 were cultured in RPMI1640 (Thermo Fisher Scientific #21875-034), supplemented with 10% heat-inactivated FBS (Gemini #CAT) and 1X antibiotic-antimycotic (Thermo Fisher Scientific #15240062). HEPG2 were cultured in DMEM (Thermo Fisher Scientific A4192001) supplemented with 10% heat-inactivated FBS (Gemini #100-500) and 1X anti-anti (Thermo Fisher Scientific #15240062). FB were cultured in DMEM/F-12 (Thermo Fisher Scientific, #11320-033), 20% FBS, 1% GlutaMAX (2 mM), 1% Pen-Strep (Gibco #15140122). Cells were plated in 384-well plates (Perkin-Elmer Cell Carrier Ultra #6057300) in 75uL media. 1000 cells/well for A549, OVCAR4, DU145, 786-0 and FB. HEPG2 were plated at 2500 cells/well.

Compound treatments

Compound treatments were added using an Echo 650 (Beckman Coulter), to a final DMSO concentration of 0.1% (75nL compound or vehicle in 75uL media). Compounds were added at either 10uM or 5uM (library dependent). Cells were incubated with compound for 48h.

Microscopy

Plates were imaged in confocal mode on the Operetta CLS high-content imaging system (Perkin Elmer) using a 20X water immersion lens (NA1.0), effective resolution (0.66um). 9 fields of view were captured per well using a 4.7MP 16-bit sCMOS sensor (6.5um pixel size).

Feature extraction

Harmony (version 4.9, Perkin Elmer) software was used to segment individual cells and extract features. First, images were flatfield-corrected and background subtracted. Next, individual nuclei were segmented using the Hoechst channel, and cytoplasm using the Alexa 568 channel (WGA, Phalloidin). Cells touching image borders were filtered out. We next calculated groups of features encompassing morphology, intensity and texture for each channel, totaling 77 features. For a full list of features, see Supporting Information Table 2.

Phenotypic profiles

Phenotypic profiles transform the measured features of single cells within a treated well into a population-level measure of the deviation from the negative controls contained in the same plate, using a non-parametric signed Kolmogorov-Smirnov (KS) statistic.[2]

Plate position normalization

Preliminary data analysis showed positional effects for a subset of plates in our dataset. That is, certain rows/columns of the plate showed systematically higher/lower feature levels among DMSO controls. These effects were also observable in PCA projections of DMSO controls (Supporting Information Fig. S3). To address these effects, we regressed phenotypic profiles against row and column IDs within each plate and subtracted out predicted values, effectively removing the portion of a phenotypic profile that could be explained by plate position (Supporting Information Fig. S1-S3). Following this regression, each feature was

centered at the median DMSO value within each plate.

Correlation feature-weighting

Our distance-based analyses assessed the similarity of samples collectively across a range of phenotypic features. To address bias introduced by highly correlated features—i.e. highly correlated features capture similar information, which will be weighted more heavily by euclidean distance as more features pick up this information—we re-weighted l2 normalized features based on the sum of their absolute correlations. That is, we re-weighted features within each cell line as

$$W_j * X_{\cdot j}/\|X_{\cdot j}\|_2, \quad W_j \propto \left(\sum_{j'=1}^{p} 1 - |C_{j,j'}| \right), \tag{1}$$

where $C_{j,j'}$ denotes the Pearson correlation between features j, j' and $X_{\cdot j}$ denotes feature vector j for all samples from a select cell line after plate position normalization.

Replicates and multiple dose levels

In the case where the experimental data contained multiple doses of a compound, only the highest dose was taken. For each compound, we averaged position-normalized, correlation-weighted KS scores across replicates at the highest dose used (when multiple doses or replicates were present in the experimental data)

Quantitative definitions of phenoactivity and phenosimilarity

For simplicity, the following notations are defined relative to a single cell line unless explicitly stated otherwise. Our full dataset can be viewed as $K = 6$ (cell lines) instantiations of the data described below, with the same compound library screened across each cell line. All distances described throughout this section are evaluated between phenotypic profiles as described above.

Let $X \in \mathbb{R}^{n \times p}$ denote phenotypic profiling data for a given cell line, with rows $X_i \in \mathbb{R}^p$ being the phenotypic profile for sample i. Samples $i = 1, ..., n$ represent different compounds from a library of interest (i.e., a single replicate of a compound perturbation, which is the same as a single well on an imaging plate). Each sample has a unique compound label $m_i \in \{1, \ldots, M\}$ and n_m denotes the number of samples labeled as MOA m. Let $I_C \subset \{1, ..., n\}$ index the DMSO control samples and $I_m \subset \{1, ..., n\}$ the samples labeled with MOA m.

Our goal is to select the optimal cell line(s) $S^* \subset 1, \ldots, K$ relative to an analytical criteria of interest. Toward this end, we consider the distance between compounds in phenotypic space, with $d_{ij} := d(X_i, X_j)$ denoting the euclidean distance between samples i, j. The approach described below can be used substituting another distance metric of interest with euclidean distance. Our criteria compare the distribution of distances from a query population of interest (e.g. compounds with the same MOA) to a reference population (e.g. DMSO controls). Comparing different query and reference populations allows us to address different questions. In the following sections, we show how optimal cell line selection for both phenoactivity and phenosimilarity can be framed within this context.

Phenoactivity

Let $\bar{X}_{I_C}$ denote the median (i.e. centroid; computed feature-wise) phenotypic profile for DMSO samples. For a population of samples, indexed by $I \subset \{1, \ldots, n\}$, consider their distances to the DMSO centroid

$$\{d(X_j, \bar{X}_{I_C}) : j \in I\}, \tag{2}$$

and denote the corresponding empirical cumulative distribution function (ECDF) of these distances as $F_{\bar{I}}$. We define the phenoactivity for a MOA m by comparing query: $I = I_m$ and

reference: $I = I_C$ populations as

$$PA_m := h(F_{\bar{I}_C}, F_{\bar{I}_m}), \tag{3}$$

where h is a function measuring the deviation ECDFs. In this study, we set h to be a signed variant of the earth mover distance that measures the difference between ECDFs

$$h(F_{\bar{I}_C}, F_{\bar{I}_m}) := \frac{2}{|\bar{I}_C \cup \bar{I}_m|} \sum_{x \in \bar{I}_C \cup \bar{I}_m} F_{\bar{I}_c}(x) - F_{\bar{I}_m}(x) \tag{4}$$

Comparing PA_m across cell lines allows us to evaluate which lines are most sensitive to MOA m (Fig 1. A-C). To evaluate phenoactivity of MOA m across sets of cell lines $S \subseteq \{1, ..., K\}$, we take the maximum phenoactivity score over all cell lines in S, effectively asking whether any of these cell lines differentiates MOA m from DMSO control. We denote phenoactivity for a set of cell lines as

$$PA_m(S) := \max_{k \in S} PA_m(k), \tag{5}$$

where $PA_m(k)$ is the phenoactivity score for MOA m in cell line k.

We cast cell line selection as an optimization problem

$$S^* := \max_{S} \sum_{m=1}^{M} w_m PA_m(S), \tag{6}$$

where $w_m \in [0, 1]$ denotes a user-provided weight associated with MOA m. In other words, our optimization criteria is simply a weighted average of phenoactivity scores across different MOAs. Weights allow us to prioritize compound classes of interest. For instance, setting weights equal will select a "generalist" cell line that performs well across all MOAs. In contrast, setting weights higher for a particular MOAs will select cell lines that are specialists within those classes.

Phenosimilarity

We assess phenosimilarity in a query MOA m by evaluating whether samples are close to one another in the MOA relative to neighboring samples in phenotypic space. These neighboring samples may have the same MOA as samples $i \in I_m$, implying that MOA m is tightly clustered in phenotypic space, or include different MOAs, implying that MOA m is intermingled with other MOAs in phenotypic space.

Consider the the pairwise distances between compounds with MOA m $\{d_{ij} : i, j \in I_m\}$ and let F_{I_m} denote the corresponding ECDF. As a baseline for this distance distribution, we use the distances between samples in I_m and their nearest neighbors as $\{d_{ij} : i \in I_m, j \in \mathcal{N}_m(i)\}$, with corresponding ECDF $F_{\mathcal{N}_m}$, where $\mathcal{N}_m(i)$ are the n_m nearest neighbors of sample i. We define the phenosimilarity for MOA m as

$$PS_m := 1 + h(F_{\mathcal{N}_m}, F_{I_m}). \tag{7}$$

We note that $F_{\mathcal{N}_m}$ provides a lower bound on F_{I_m} in the sense that $h(F_{\mathcal{N}_m}, F_{I_m}) \leq 0$, with equality only when all nearest neighbors belong to MOA m. Thus (7) returns a values between 0 and 1 when all nearest neighbors are from MOA m; values < 1 represent varying degrees of overlap with other MOAs.

For a set of cell lines S, we define MOA similarities as the maximum across all cell lines $k \in S$, denoted as $PS_m(S)$. This asks whether any cell line $k \in S$ groups MOA m compounds. As in the case of phenoactivity, we cast cell line selection for phenosimilarity as an optimization problem

$$S^* := \max_S \sum_m w_m * PS_m(S). \tag{8}$$

References

(1) Kang, J.; Hsu, C.-H.; Wu, Q.; Liu, S.; Coster, A. D.; Posner, B. A.; Altschuler, S. J.; Wu, L. F. Improving drug discovery with high-content phenotypic screens by systematic selection of reporter cell lines. *Nature biotechnology* **2016**, *34*, 70–77.

(2) Perlman, Z. E.; Slack, M. D.; Feng, Y.; Mitchison, T. J.; Wu, L. F.; Altschuler, S. J. Multidimensional drug profiling by automated microscopy. *Science* **2004**, *306*, 1194–1198.

Supporting Information

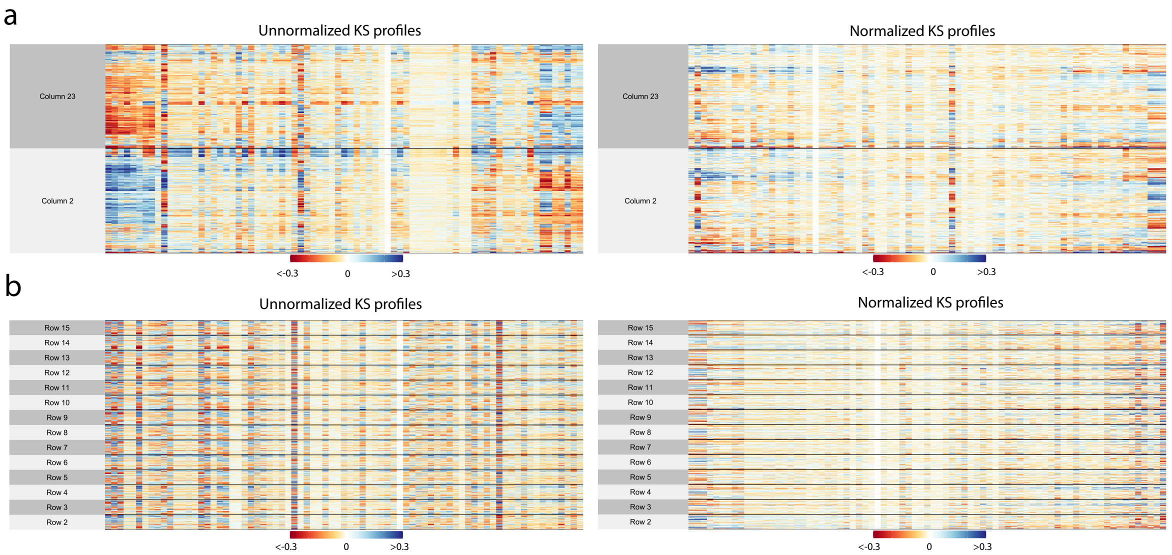

Figure S1: KS profiles of each DMSO sample before (left) and after (right) plate position normalization. Rows: well replicates, columns: image-derived features. **(a)** samples are grouped by column on imaging plate. **(b)** samples are grouped by row on imagining plate.

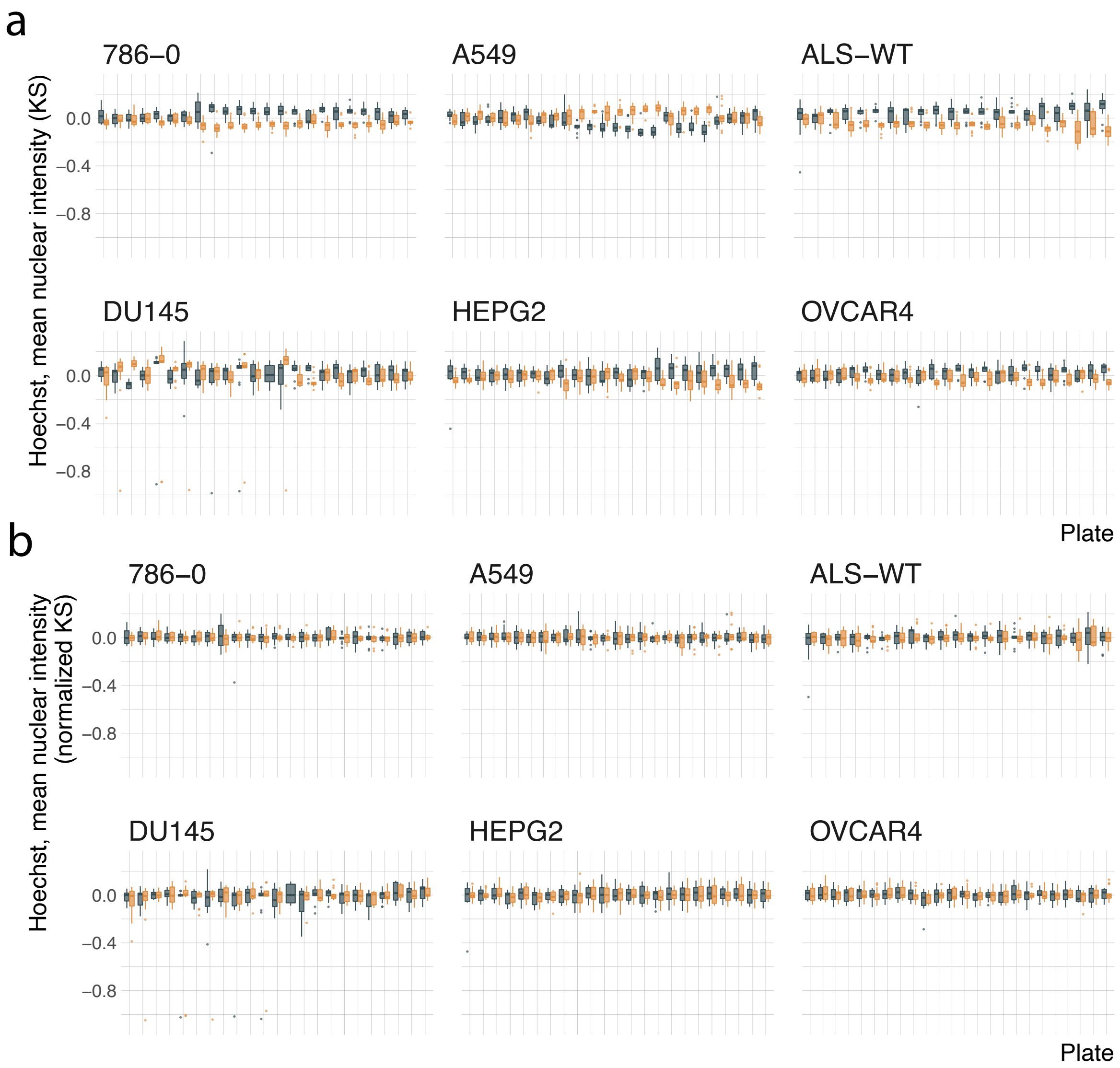

Figure S2: Distribution of hoechst nuclear intensity in DMSO samples before **(a)** and after **(b)** plate position normalization by plate. Color: column on imaging plate (grey = 23, yellow = 2).

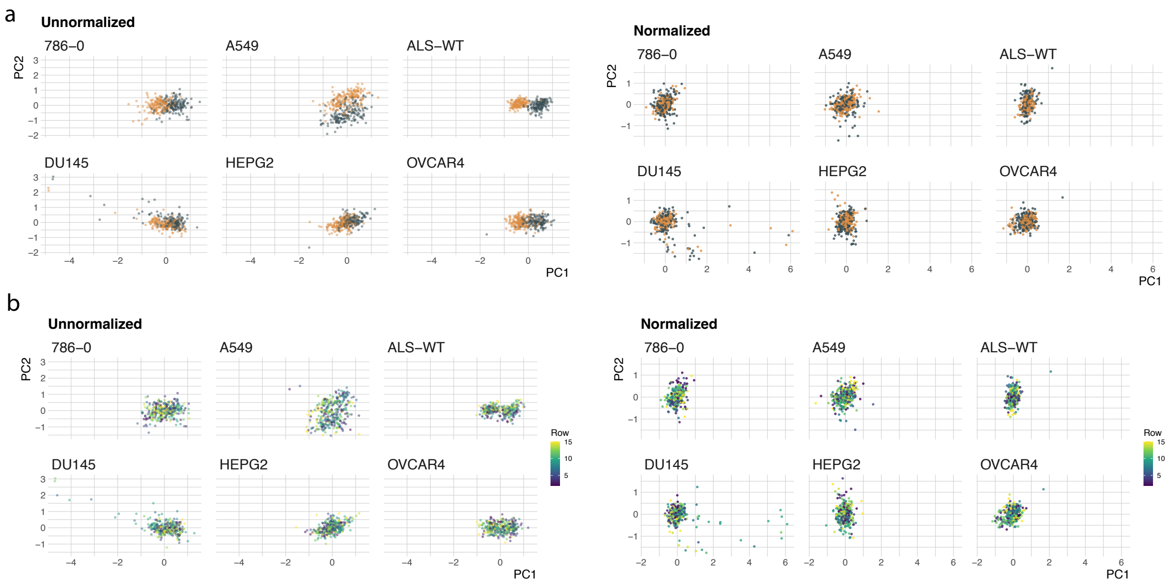

Figure S3: PCA projections of DMSO samples before (left) and after (right) plate position normalization. **(a)** Color: column on the imaging plate (grey = 23, yellow = 2). **(b)** Color: row on the imaging plate.

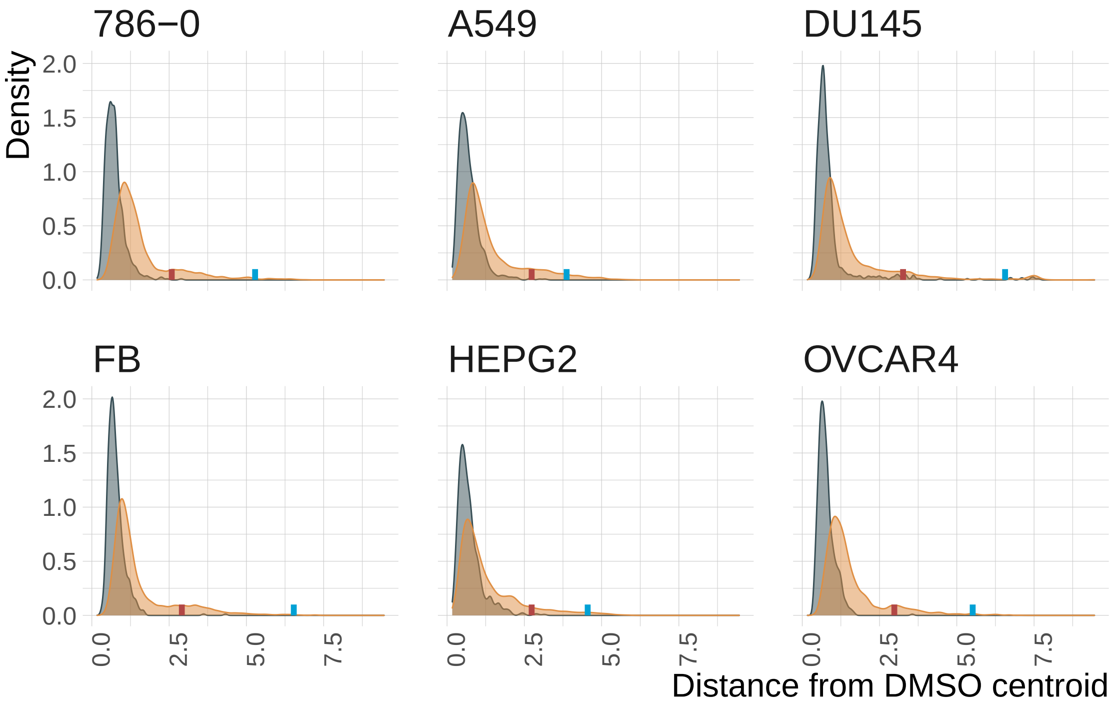

Figure S4: Distance to DMSO point cloud centroid by cell line. Lines indicate the distance to DMSO centroid for positive control compounds. Colors: grey = DMSO samples, yellow = query compounds; red line = Gemcitabine, blue line = Bortezomib.

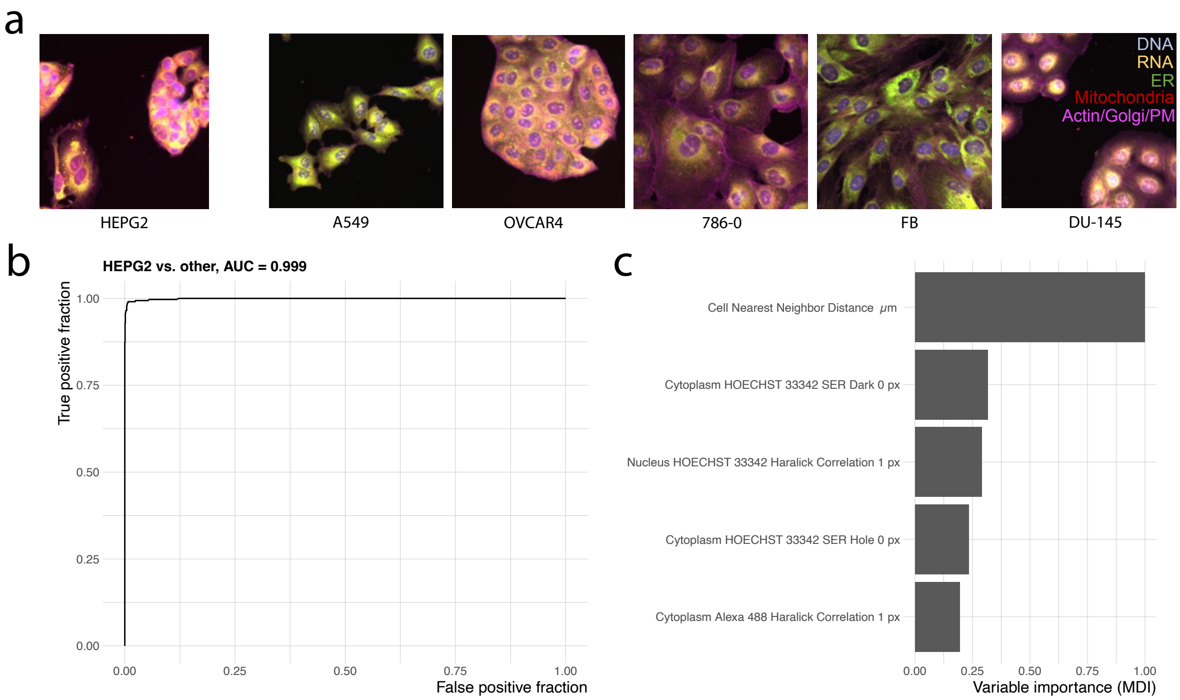

Figure S5: HEPG2 versus other cell lines. **(a)** Representative images of cells after 48 hours exposure to DMSO vehicle control 0.1%. Scale bar represents 100um. **(b)** ROC curve of iterative random forest model trained to classify HEPG2 versus other cell lines. **(c)** MDI feature importance of top 5 features used in HEPG2 versus other cell line classifier.

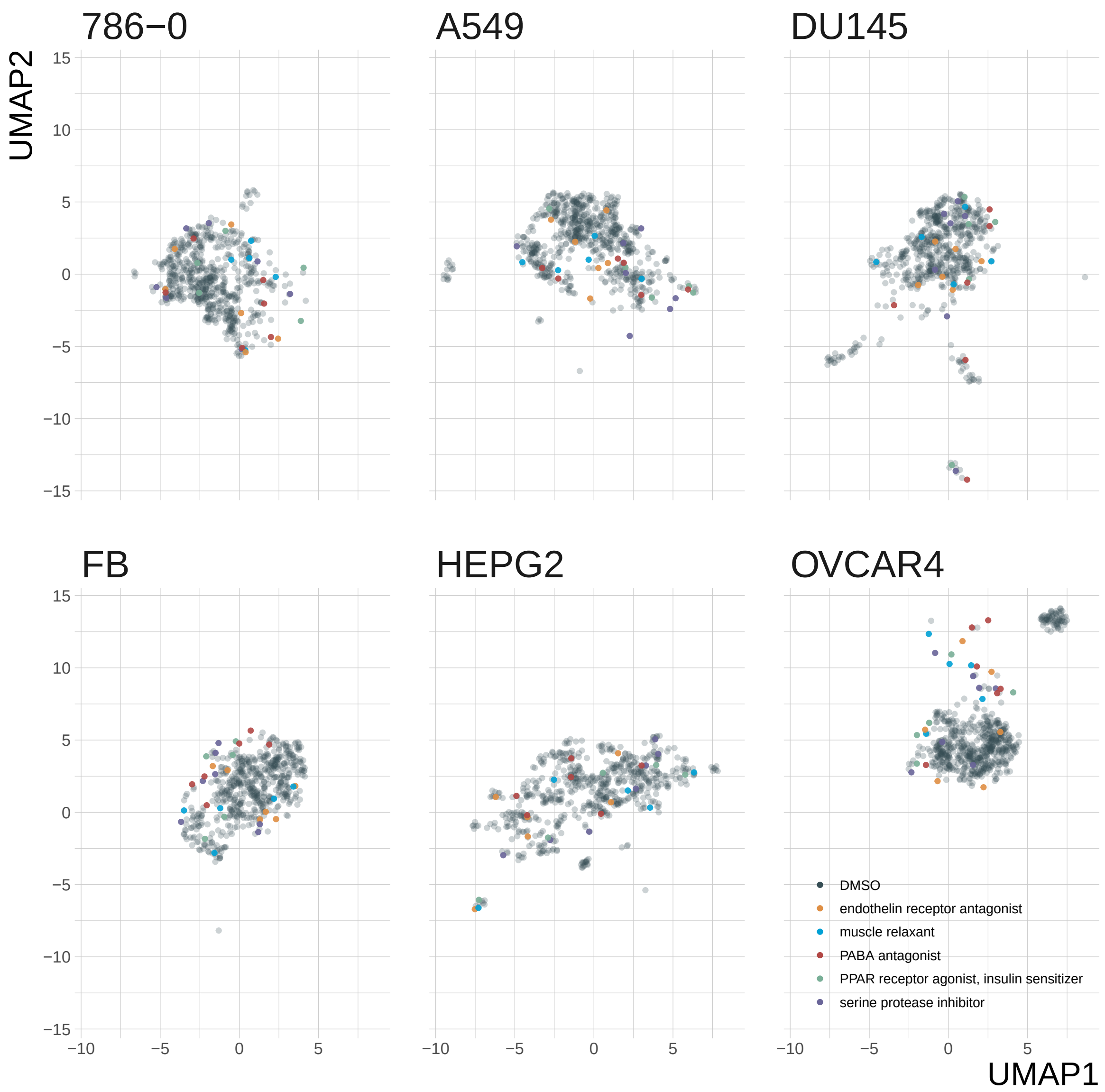

Figure S6: UMAP projections of MOAs with low phenosimilarity across all cell lines and DMSO controls. MOAs selected based on lowest rank of maximum phenosimilarity across all cell lines

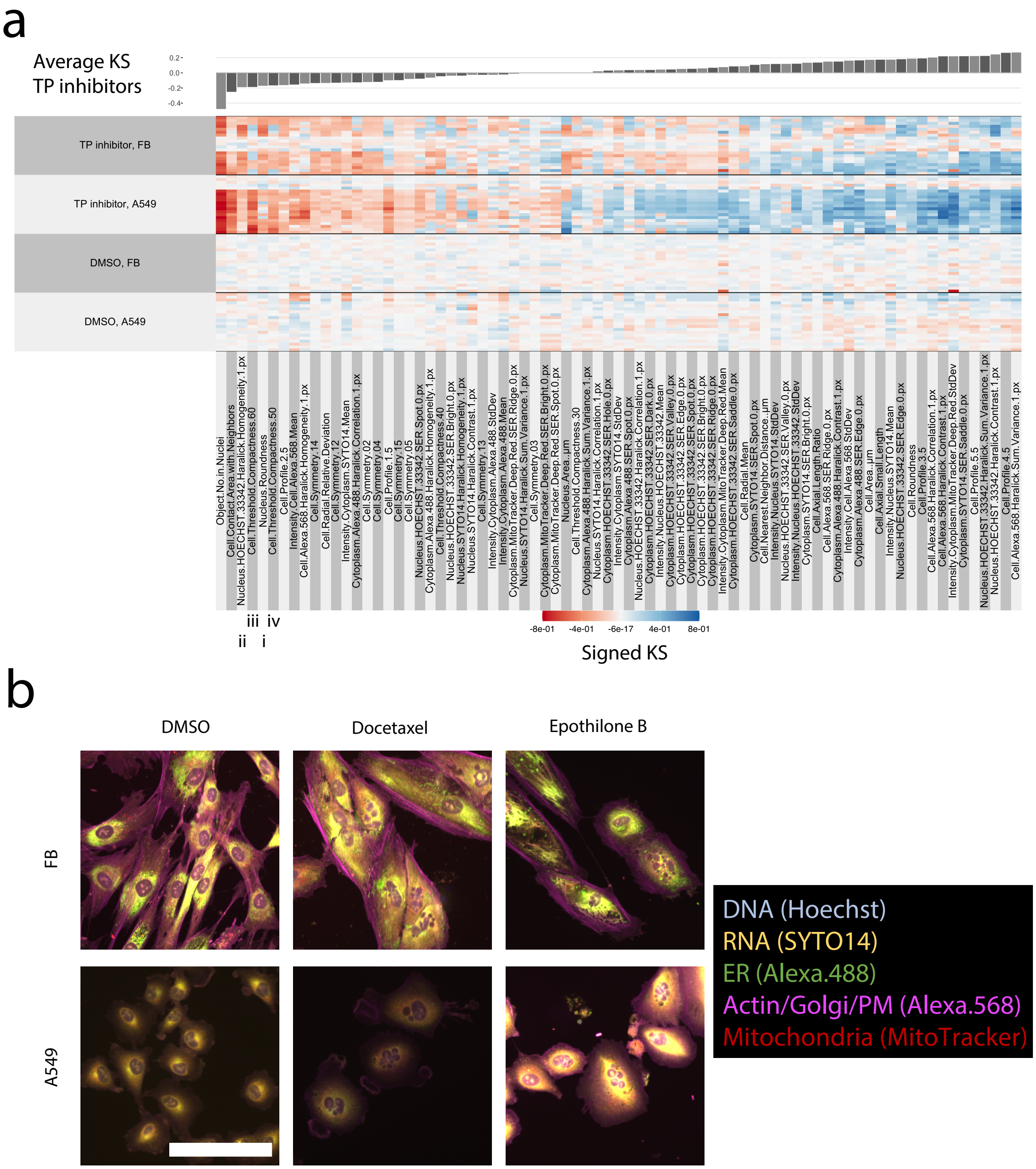

Figure S7: Phenotypic profiles **(a)** and representative images **(b)** for TP inhibitors. Features ordered based on average (across compounds and cell lines) signed KS value in TP inhibitors. Images of vehicle (DMSO 0.1%) and compound treated cells (A540 or FB) after 48 hours exposure. Scale bar represents 100um. Indicators i-iv highlight features referenced in the text.

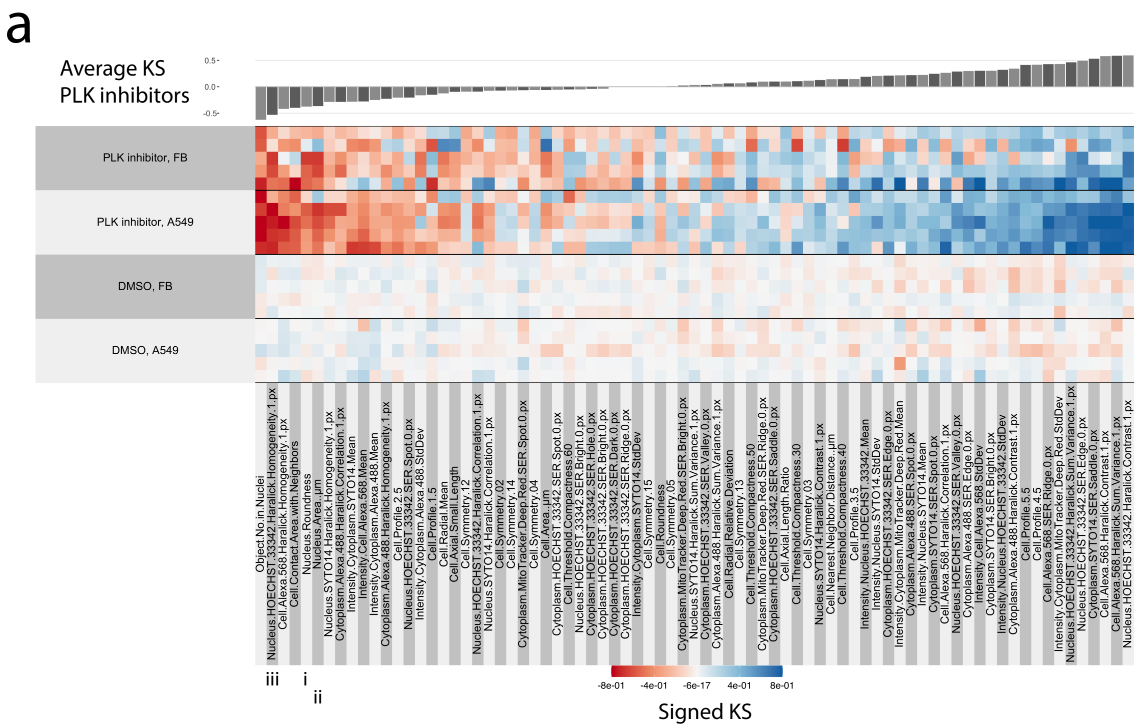

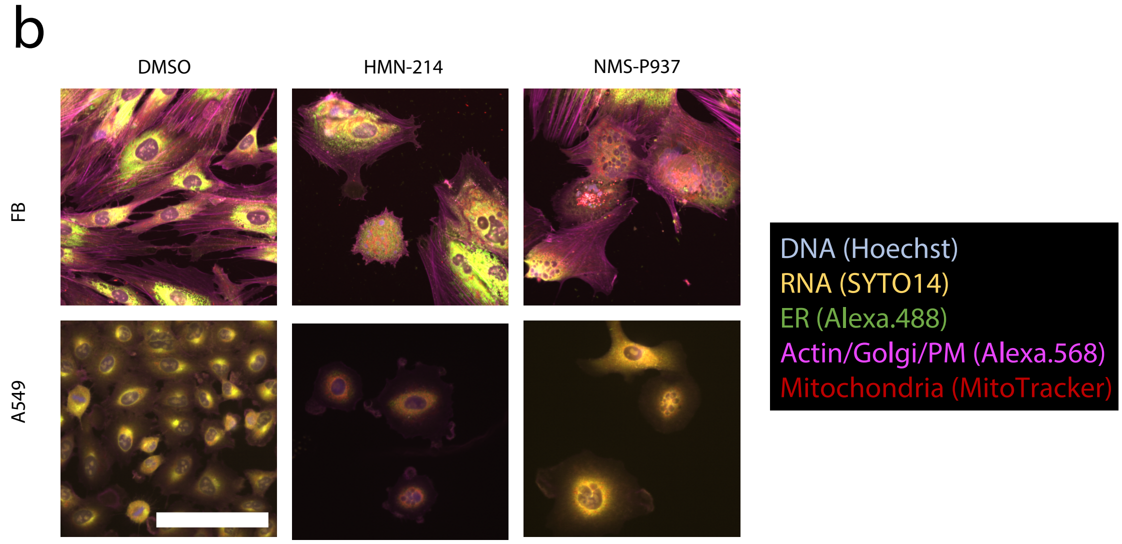

Figure S8: Phenotypic profiles **(a)** and representative images **(b)** for PLK inhibitors. Features ordered based on average (across compounds and cell lines) signed KS value in PLK inhibitors category. Images of vehicle (DMSO 0.1%) and compound treated cells (A540 or FB) after 48 hours exposure. Scale bar represents 100um. Indicators i-iii highlight features referenced in the text.

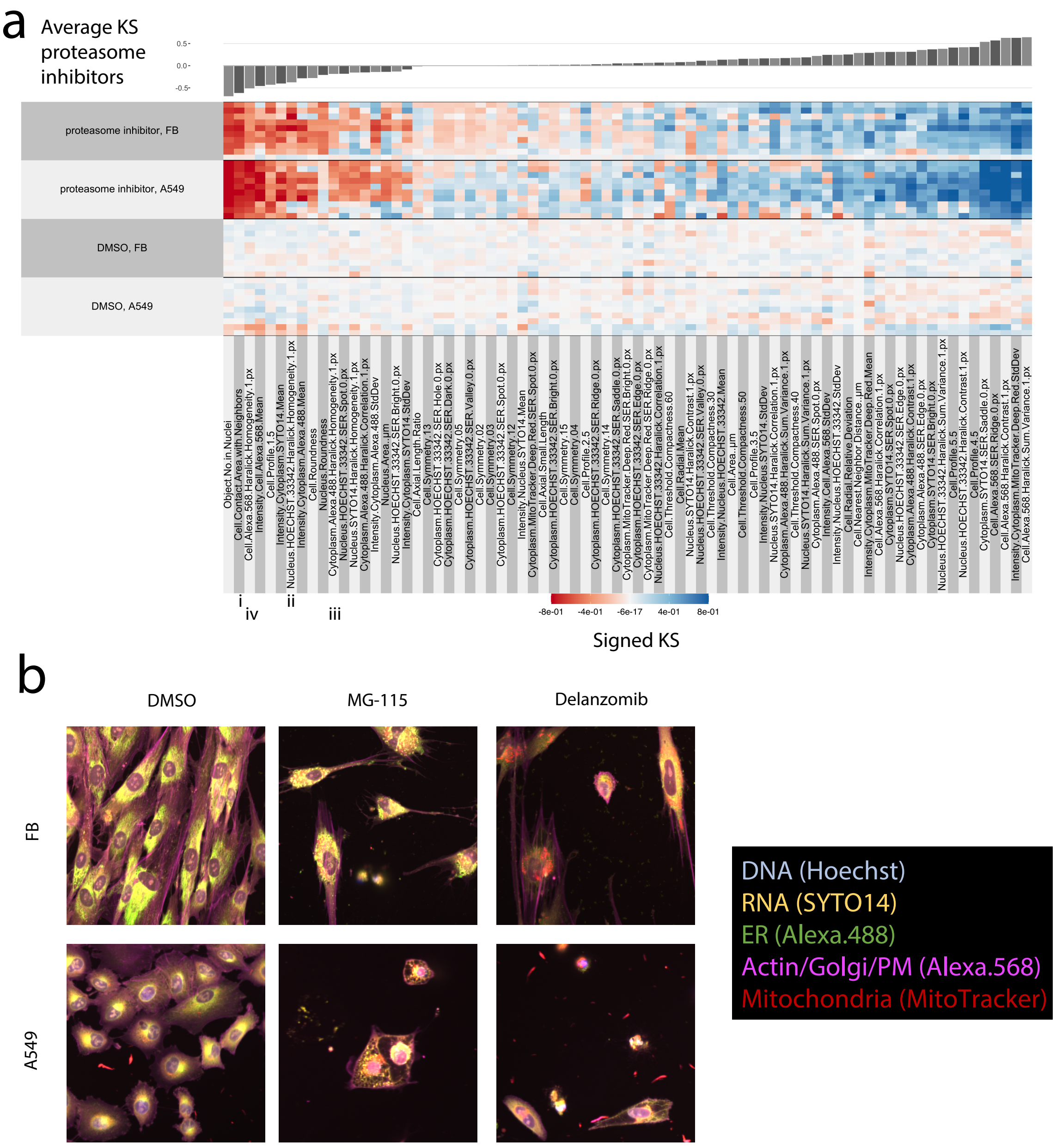

Figure S9: Phenotypic profiles **(a)** and representative images **(b)** for proteasome inhibitors. Features ordered based on average (across compounds and cell lines) signed KS value in proteasome inhibitors category. Images of vehicle (DMSO 0.1%) and compound treated cells (A540 or FB) after 48 hours exposure. Scale bar represents 100um. Indicators i-iv highlight features referenced in the text.
